# Inhibition of ERAD synergizes with FTS to eradicate pancreatic cancer cells

**DOI:** 10.1186/s12885-021-07967-6

**Published:** 2021-03-06

**Authors:** Rong Du, Delaney K. Sullivan, Nancy G. Azizian, Yuanhui Liu, Yulin Li

**Affiliations:** 1 Center for Immunotherapy Research, Houston Methodist Research Institute, Houston, TX 77030 USA; 2 Department of Medicine, Weill Cornell Medical College, New York, NY 10065 USA; 3 Department of Neurosurgery, Xiangya Hospital, Central South University, Changsha, 410008 Hunan China; 4 UCLA-Caltech Medical Scientist Training Program, David Geffen School of Medicine, University of California, Los Angeles, CA 90095 USA

**Keywords:** Pancreatic ductal adenocarcinoma (PDAC), Clusters of regularly interspaced short palindromic repeats (CRISPR), Farnesyl thiosalicylic acid (FTS), Salirasib, Endoplasmic reticulum-associated protein degradation (ERAD), Unfolded protein response (UPR)

## Abstract

**Background:**

Pancreatic ductal adenocarcinoma (PDAC), one of the most lethal cancers, is driven by oncogenic KRAS mutations. Farnesyl thiosalicylic acid (FTS), also known as salirasib, is a RAS inhibitor that selectively dislodges active RAS proteins from cell membrane, inhibiting downstream signaling. FTS has demonstrated limited therapeutic efficacy in PDAC patients despite being well tolerated.

**Methods:**

To improve the efficacy of FTS in PDAC, we performed a genome-wide CRISPR synthetic lethality screen to identify genetic targets that synergize with FTS treatment. Among the top candidates, multiple genes in the endoplasmic reticulum-associated protein degradation (ERAD) pathway were identified. The role of ERAD inhibition in enhancing the therapeutic efficacy of FTS was further investigated in pancreatic cancer cells using pharmaceutical and genetic approaches.

**Results:**

In murine and human PDAC cells, FTS induced unfolded protein response (UPR), which was further augmented upon treatment with a chemical inhibitor of ERAD, Eeyarestatin I (EerI). Combined treatment with FTS and EerI significantly upregulated the expression of UPR marker genes and induced apoptosis in pancreatic cancer cells. Furthermore, CRISPR-based genetic ablation of the key ERAD components, HRD1 and SEL1L, sensitized PDAC cells to FTS treatment.

**Conclusion:**

Our study reveals a critical role for ERAD in therapeutic response of FTS and points to the modulation of UPR as a novel approach to improve the efficacy of FTS in PDAC treatment.

**Supplementary Information:**

The online version contains supplementary material available at 10.1186/s12885-021-07967-6.

## Background

The most prevalent pancreatic malignancy, pancreatic ductal adenocarcinoma (PDAC), is among the most aggressive cancers, with a dismal prognosis, and highly refractory to most treatment options [1]. KRAS mutations have been identified as the oncogenic driver in PDAC [2]. RAS is a membrane bound GTPase that functions as a molecular switch, cycling between active GTP-bound state facilitated by guanine nucleotide exchange factors, and inactive GDP-bound state regulated by GTPase-activating proteins [3]. Upon activation of cell-surface receptors, RAS switches to the GTP-bound state to recruit and activate cytoplasmic effectors involved in a variety of signaling networks [4]. Oncogenic mutations impair the ability of KRAS to hydrolyze GTP, and lock the oncoprotein in its constitutively active GTP-bound state, leading to hyperactivation of effector signaling pathways driving tumorigenesis [5].

Given its critical function as an oncogenic driver, extensive efforts have been devoted to the development of specific RAS inhibitors [6–8]. However, the lack of binding sites for small chemical inhibitors on the RAS protein surface has hindered the efforts to directly target RAS. Furthermore, drugging RAS through the use of GTP antagonists has not been feasible due to the extremely high affinity (picomolar) to GTP and GTP abundance (millimolar) in cells. Failure to develop clinically-approved RAS inhibitors has prompted the perception that RAS proteins are “undruggable” [9]. In recent years, exciting progresses have been made in directly targeting the rare KRAS^G12C^ mutant [10–14], but not the more prevalent mutations, such as G12D, G12V, and G12R.

RAS proteins undergo a series of post-translational modifications related to their biological functions such as acetylation, ubiquitination, phosphorylation, and farnesylation among many [15]. Localization of KRAS on plasma membrane is crucial to downstream signal transduction, and farnesylation at C-terminal cysteine (CAAX motif) is essential for its localization and activation [16]. Farnesyl thiosalicylic acid (FTS), also known as salirasib, was developed as a pan-RAS inhibitor [17]. FTS mimics the C-terminal farnesyl cysteine carboxymethyl ester of all RAS isoforms and selectively dislodges active RAS proteins from cell membrane, inhibiting their biological functions [18]. Promising findings in preclinical models have prompted several clinical trials in leukemia, lung, and pancreatic cancers [19, 20]. However, FTS alone has shown only modest activity in patients despite minimal side effects [21–23], suggesting further efforts to develop FTS for the treatment of PDAC should be focused on drug combinations to enhance the therapeutic efficacy.

Combinatorial strategies, such as coupled targeting of KRAS and mitochondrial respiration pathways [24], or concomitant suppression of ERK and autophagy [25], have been proposed for PDAC treatment with the goal of achieving better therapeutic responses. The concept of synthetic lethality has been widely exploited in the development of targeted therapeutics and combination therapies. The conceptual framework posits that, two perturbations are considered synthetic lethal if either one acting alone is compatible with viability, but when combined, will lead to cell death [26, 27]. Functional genetic approaches, such as RNAi and CRISPR screens, have been employed to discover novel genetic targets of synthetic lethality and identify combination therapies [28, 29]. In particular, CRISPR-Cas9 genome-wide screens have superior efficiency and increased specificity [30, 31]. We, therefore, set out to identify genetic targets that enhance FTS efficacy in PDAC by performing a genome-wide CRISPR synthetic lethality screen. Among the top candidates, genes related to endoplasmic reticulum-associated protein degradation (ERAD) were highly enriched. Moreover, we showed that FTS induced unfolded protein response (UPR) in murine and human PDAC cells, which was further enhanced by suppression of ERAD to induce apoptosis.

## Methods

### Cell lines and chemical reagents

4292 murine PDAC cell line, expressing mutant KRAS^G12D^ under the control of the Tet-ON system, and p53^R172H^, was a generous gift from Dr. Marina Pasca di Magliano at the University of Michigan [32, 33]. 4292 cells were cultured in RPMI 1640 supplemented with 10% FBS, 1% penicillin/streptomycin, and 1 μg/ml doxycycline. Human PDAC cell lines, PANC-1 and MIA PaCa-2, were purchased from ATCC and cultured in DMEM supplemented with 10% FBS and 1% penicillin/streptomycin. FTS, Eeyarestatin I, and GSK2606414 were purchased from Cayman Chemical.

### Pooled “synthetic lethal” CRISPR screen and data analysis

The CRISPR library screen was performed following published protocols [34]. The pooled mouse CRISPR lentiviral library containing 78,637 gRNAs targeting 19,674 genes was obtained from Addgene (#73633-LV, lentiviral prep), and the screen was performed in duplicate with two independent viral infections. Briefly, 4292 cells with stable Cas9 expression were infected at low viral titers so that approximately 10% of the cells were infected. The infected cells were selected with puromycin (1 μg/ml) for 2 days. The experimental pool was treated with 100 μM FTS for 12 days. Both experimental and control pools were passaged every 3 days with at least 50 million cells per pool to ensure 500-1000X coverage. Following FTS treatment, the gRNA libraries in the cell populations were isolated by PCR amplification and identified by Hiseq. Computational analysis of the libraries was performed using MAGeCK (version 0.5.9.2). First, cutadapt (version 2.6) was used to trim primer and other technical sequences from paired-end FASTQ files with the specified options: -j 0 --maximum-length 30 --pair-filter = both -e 0.3 -g GCTTTATATATCTTGTGGAAAGGACGAAACACC…GTTTTAGAGCTAGAAATAGCAAGTTAAAATAAGGCTAGTCCGTTATCAACTTGAAAAAGT -G CCGACTCGGTGCCACTTTTTCAAGTTGATAACGGACTAGCCTTATTTTAACTTGCTATTTCTAGCTCTAAAAC…GGTGTTTCGTCCTTTCCACAAGATATATAAAGC. Second, the command, mageck count, was run under default options with the trimmed paired-end FASTQ files supplied in the arguments --fastq and --fastq-2 to collect read counts. Finally, the command, mageck mle, was run, specifying an unpaired design for the biological duplicates, 10 permutation rounds, 2500 genes for mean-variance modeling, the --remove-outliers option, median normalization, FDR *p*-value adjustment, and a list of the 1000 control sgRNAs in the library supplied to the --control-sgrna option. The resulting beta scores and FDR-adjusted permutation *p*-values were used in downstream analysis. All samples had a mapping rate of 89–90.2%, gini index (a measure of the evenness of sgRNA read counts) of 0.066–0.072, and 384–502 zero-count sgRNAs. Enrichr was used for gene ontology (GO) analysis of genes with a negative beta score and an FDR < 0.05 (*n* = 488 genes) [35]. The visualization tool, Clustergrammer, was used to graphically depict the top 20 GO terms (as ranked by *p*-value) and their associated genes.

### IC50 determination and cell viability assay

Cells were seeded at the following concentrations: 4000 cells for 4292, 16,000 cells for PANC-1, 8000 cells for MIA PaCa-2 per well in 24-well plates. Drugs were added the next day in triplicates. Following 5 days of drug treatment, cell viability was assessed using the CellTiter-Glo 2.0 Cell Viability Assay (Promega) according to the manufacturer’s instructions. Data analysis and calculation of the half-maximal inhibitory concentration (IC50) were carried out using GraphPad Prism 8 (GraphPad).

### Western blot analysis

Cells were lysed on ice in CelLytic MT Cell Lysis Reagent (Sigma) supplemented with protease and phosphatase inhibitors. Protein concentration was determined with Pierce BCA Protein Assay Kit (Thermo Fisher). Protein lysates were resolved on SDS-PAGE gels and transferred to PVDF membranes [36]. The following antibodies were used for the western blot analysis: phospho-eIF2α (#9721, 1:1000), eIF2α (#9722, 1:1000), ATF4 (#11815, 1:1000), BIM (#2819, 1:1000), SYVN1 (#14773, 1:1000) from Cell Signaling Technology; phospho-PERK (Thr980) (MA5–15033, 1:1000, Thermo Fisher); SEL1L (S3699, 1:1000, Sigma); β-tubulin (10094–1-AP, 1:5000, Proteintech).

### RNA extraction and quantitative real-time PCR

RNA was extracted with the RNeasy Mini Kit (Qiagen). cDNA was synthesized using the Verso cDNA Synthesis Kit (Thermo Scientific) according to the manufacturer’s instructions. qPCR analysis was performed on a 7500 Real-Time PCR System using SYBR Green (Applied Biosystems). Primer sequences are available in Supplementary Table 3. Transcripts were normalized to human GAPDH or mouse UBC, and relative gene expression was determined with ddCt method as previously reported [36].

### Flow cytometry

PE Annexin-V Apoptosis Detection Kit (BD Biosciences) was used to detect apoptosis following manufacturer’s instruction [36]. Briefly, cells were suspended in 100 μl of 1X binding buffer and mixed with 5 μl of PE-conjugated annexin-V and 7-AAD respectively, followed by incubation at room temperature for 15 min in the dark. The stained cells were analyzed using a BD FACS Fortessa flow cytometer (BD Bioscience). All flow cytometric data were analyzed with FlowJo software (Tree Star).

### CRISPR sgRNA generation, transfection and single-cell sorting

For construction of sgRNA-expressing vectors, DNA oligonucleotides were annealed and cloned in BbsI-digested pSpCas9(BB)-2A-GFP (PX458) plasmid (Addgene #48138). Target sgRNA oligonucleotide sequences are listed in Supplementary Table 4. MIA PaCa-2 cells were seeded in 6-well plates at 0.5 million/well the day before transfection. PX458 empty vector or pooled sgRNA plasmids (2.5 μg/well) were transfected into MIA PaCa-2 cells using lipofectamine 3000 (Invitrogen). Three days after transfection, cells were collected and GFP-positive cells were sorted in 96-well plate using a BD FACS Fortessa flow cytometer (BD Bioscience).

### Retrovirus packaging and transduction

Retroviral vectors for the expression of fluorescent markers were combined with pCL-10A1 retrovirus packaging vector at 1:1 ratio and transfected into HEK293FT cells using lipofectamine 3000 transfection reagent (Invitrogen). Supernatants containing the viral particles were collected at 48 h post-transfection and passed through a sterile 0.45 μm syringe filter (VWR). Wild type, *SYVN1* KO1, and *SEL1L* KO1 cells were infected by viral supernatant supplemented with 6 μg/ml polybrene in 6-well plate by centrifugation at 1000 g for 90 min. Infected cells were selected by growing in DMEM medium containing 3 μg/ml puromycin for 2 days.

### Multicolor competition assay

pBabe puro IRES-mCherry (Addgene #128038) and pBabe puro IRES-EGFP (Addgene #128037) were used for labeling. MIA PaCa-2-derived single clones labeled with mCherry (wild type) and GFP (*SYVN1* KO1 or *SEL1L* KO1) were mixed at 1:1 ratio. Mixed cell populations were seeded in T25 flasks at 0.2 million cells per cell line. One day after seeding (D0) wells were imaged with Leica DMi8 with a 5X objective and mCherry and GFP positive cells were counted from three different images. Cells were treated with DMSO or FTS for 2 weeks. Relative cell fitness was calculated from the percentage of GFP-positive cells normalized to the D0 value.

### Statistical analysis

Cell viability and apoptotic cell percentage among different treatment groups were analyzed using ANOVA with Tukey’s test. Other viability assays and transcript levels were analyzed using two-tailed student’s t test. Statistical significance of multicolor competition assay was assessed using unpaired two-sample t test. Results were presented as mean ± standard deviation (SD).

## Results

### CRISPR screen identifies ERAD as a cellular process modulating FTS efficacy

**Fig. 1:**
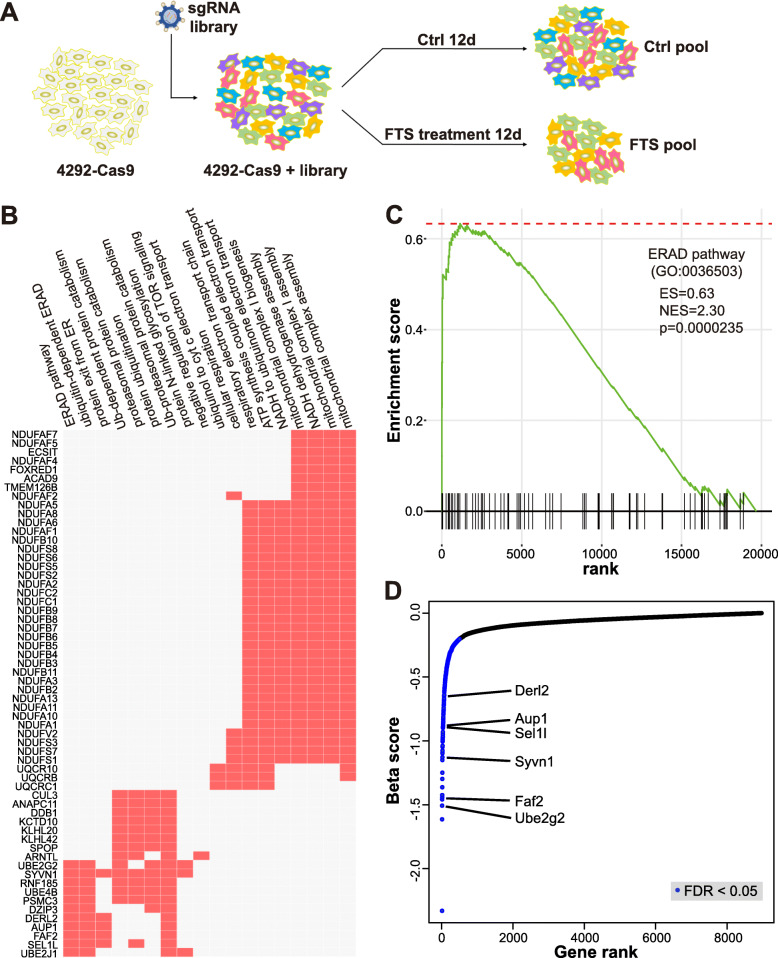
CRISPR screening identifies ERAD as a target modulating the efficacy of FTS. **a** Basic flow of the genome-wide CRISPR screen for genes modulating FTS sensitivity. **b** Gene Ontology (GO) pathway analysis of genes with gRNA dropouts upon FTS treatment. **c** Gene Set Enrichment Analysis (GSEA) showing enrichment of genes in the ERAD pathway indicated by the enrichment score and *p*-value. **d** Gene dropout rank based on beta score. Top ERAD pathway genes were labeled. Blue color depicted gene dropouts with FDR < 0.05

To discover potential genetic targets that modify sensitivity to FTS, we performed a genome-wide CRISPR library screen utilizing a murine pooled sgRNA library [37]. Cas9 nuclease was introduced in the murine PDAC cell line 4292 by lentiviral infection, followed by the sgRNA library, which contains 78,637 gRNAs targeting 19,674 genes. Following puromycin selection, the cell population containing the gRNA library was further expanded and split into control and FTS pools. FTS pool was treated with the drug for 12 days, while the control pool was maintained in culture. At the end of the treatment, genomic DNA from both pools was collected for PCR amplification of sgRNA libraries and further analysis of sgRNA frequency by deep-sequencing (Fig. 1a). A “beta score” was calculated for each gene to measure the degree of selection using MAGeCK’s maximum likelihood estimation algorithm (Supplementary Table 1) [38]. The beta score quantifies the effect size with negative values suggesting sgRNA depletion and positive values suggesting sgRNA enrichment [38].

To identify mutations that specifically enhance FTS efficacy, we searched for sgRNAs that were further depleted (with a negative beta score) upon FTS treatment. Gene Ontology (GO) pathway analysis based on false discovery rate (FDR) adjusted *p*-values of less than 0.05 pointed to several pathways in regulating the FTS response, including mitochondrial respiratory chain, protein retrograde transport from ER to cytosol, and proteasome-mediated ubiquitin-dependent protein catabolic processes (Fig. 1b**,** Supplementary Table 2). In particular, sgRNAs targeting various ERAD-related genes, including *Ube2g2*, *Faf2*, *Syvn1*, *Sel1l*, *Aup1*, and *Derl2*, were significantly depleted from the FTS pool (Fig. 1c-d) [39, 40]. These molecules are essential components of ERAD, a process responsible for the clearance of misfolded proteins in the ER through cytosolic translocation and proteasomal degradation [41]. Retro-translocation of ERAD substrates across the ER membrane is mediated by the HRD1-SEL1L complex, representing the most conserved ERAD machinery. The E3 ligase HRD1 (encoded by *Syvn1*) forms a translocation channel while SEL1L functions as the adaptor protein [42, 43]. A member of Derlin family of proteins, DERL2, is an essential functional partner of HRD1-SEL1L complex [44]. Another ERAD-related protein, AUP1, recruits the E2 ubiquitin-conjugating enzyme UBE2G2 during the ubiquitination of substrates [45]. Following retro-translocation and polyubiquitination, substrates are extracted from the ER membrane by the AAA ATPase VCP/p97 complex and transferred to the cytosolic proteasome for degradation [46, 47]. Another component, UBXD8 (encoded by *Faf2*) is an ER membrane-embedded recruitment factor for VCP/p97 [48]. Thus, our CRISPR screen identifies multiple key components of ERAD, pointing to ERAD as a major pathway affecting the therapeutic efficacy of FTS.

### ERAD inhibition synergizes with FTS to activate ER stress and induce apoptosis

**Fig. 2:**
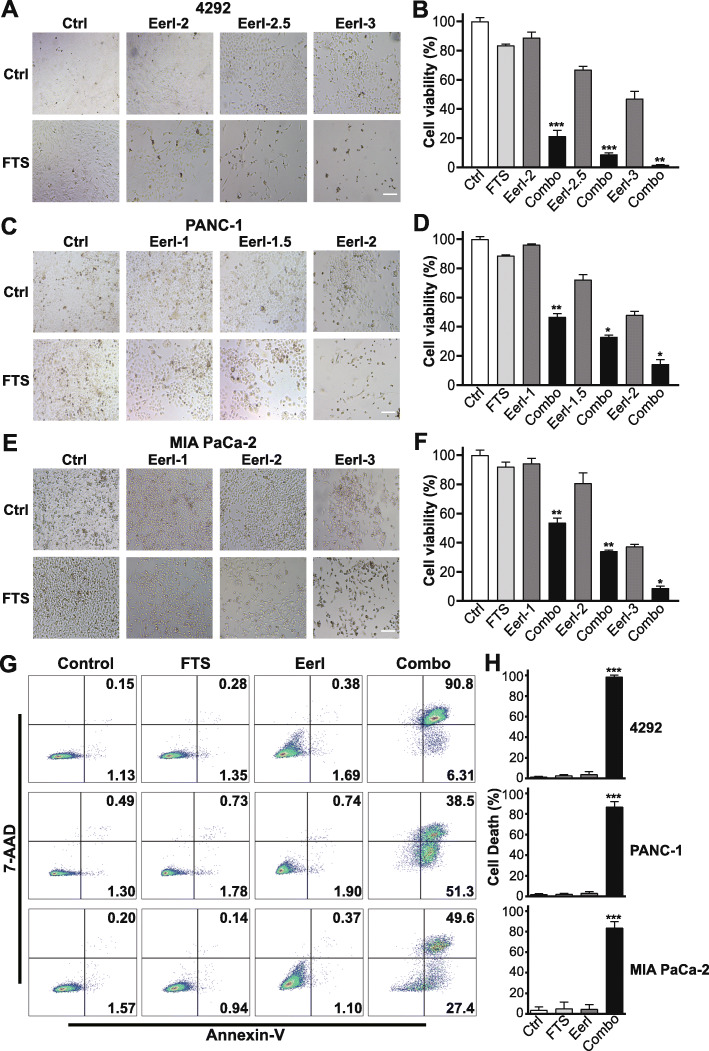
EerI synergizes with FTS to kill PDAC cells through apoptosis induction. **a**, **c**, **e** Bright field images (200X) of 4292, PANC-1, and MIA PaCa-2 cells treated with 100 μM FTS, EerI, or both for five days. EerI (μM) concentrations were indicated above each figure. Scale bar, 25 μm. **b**, **d**, **f** Relative cell viability following five days of treatment with FTS, EerI, or both. ANOVA with Tukey’s test compares the viability difference between combination and single treatments or controls. *P < 0.05, **P < 0.01, ***P < 0.001; data are presented as mean ± SD; *n* = 3). **g** Representative flow cytometric analysis of PDAC cell apoptosis following treatments with 100 μM FTS, EerI (3 μM for 4292, 2.5 μM for PANC-1 and MIA PaCa-2), or both for five days. Cells were stained with annexin-V and 7-AAD. Percentages of apoptotic cells were indicated in the quadrants. **h** PDAC cell death analysis following different treatments with Annexin V/7-AAD apoptosis assay. ANOVA with Tukey’s test compares difference treatment groups. ***P < 0.001; data presented as mean ± SD; *n* = 3.

To determine whether inhibition of ERAD could synergize with FTS treatment to induce cell death in PDAC cells, we combined an ERAD inhibitor, Eeyarestatin I (EerI), with FTS. EerI acts through the inhibition of VCP/p97, which mediates the translocation of misfolded proteins from ER to cytosol for proteasomal degradation [49, 50]. We treated three PDAC cell lines (murine 4292, human PANC-1 and MIA PaCa-2) with FTS, EerI, or both for 5 days, and assessed cell survival by microscopic observation and viability assay. While FTS only modestly reduced the viability of PDAC cells, combined treatment with EerI and FTS drastically decreased cell viability (Fig. 2). Berenbaum combination index (CI) [51] showed CI values of less than 1 for all the cell lines, confirming a synergistic effect on combined treatment (Supplementary Fig. S1).

**Fig. 3:**
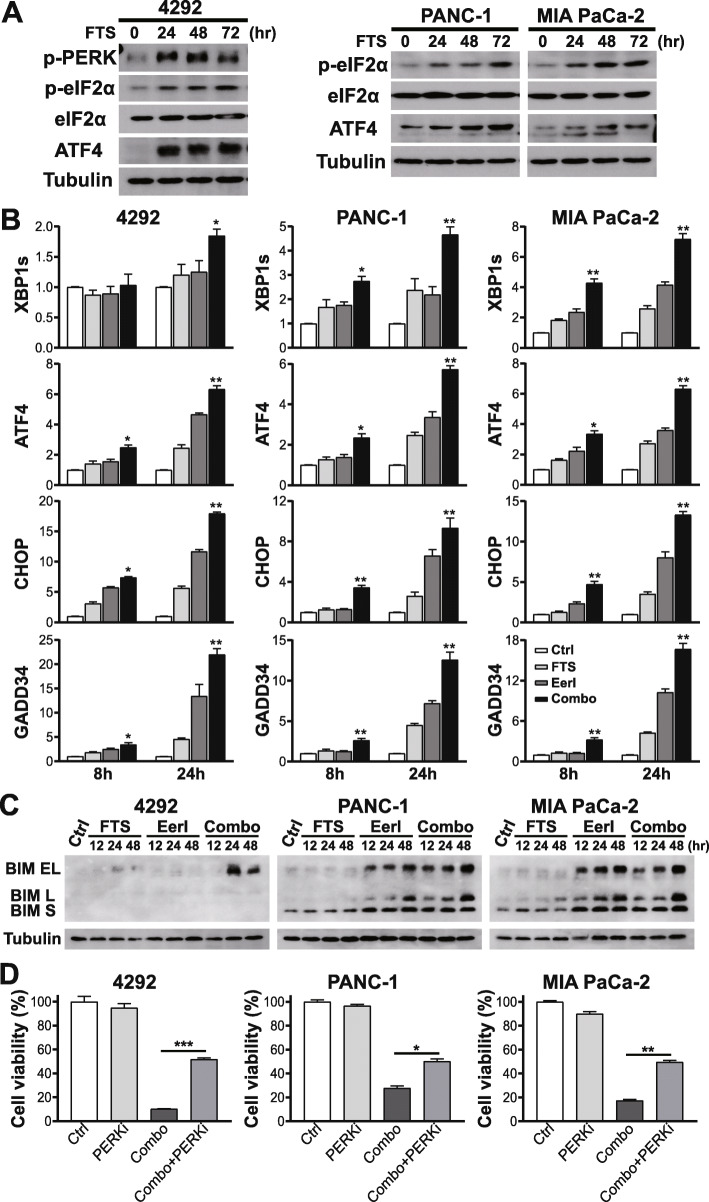
Activation of UPR by FTS is further enhanced upon ERAD inhibition to elicit ER stress-induced apoptosis. **a** Protein expression of UPR markers (p-PERK, p-eIF2α, ATF4) in PDAC cells treated with 100 μM FTS for 72 h. **b** Changes in transcript levels of UPR marker genes (XBP1s, ATF4, CHOP, and GADD34) following treatment with 100 μM FTS, EerI (3 μM for 4292 and MIA PaCa-2, 2 μM for PANC-1), or both for 8 and 24 h. All mRNA values were normalized to human GAPDH or mouse UBC. Two-tailed student’s t test compares transcriptional difference between combination and single treatments or control at the same timepoint, *P < 0.05, **P < 0.01; data presented as mean ± SD; *n =* 3. **c** Protein expression of BIM isoforms in PDAC cells in response to treatments with FTS, EerI, or the combination for 48 h. **d** Pretreatment with PERKi (2.5 μM GSK2606414) for two hours partially blocked the cell death induced by the combined FTS and EerI treatment for five days. Two-tailed student’s t test, *P < 0.05, **P < 0.01, ***P < 0.001; relative cell viability are presented as mean ± SD; *n =* 3

Tumor cells are exposed to intrinsic and environmental factors, such as oncogene activation, hypoxia, glucose deprivation, and lactic acidosis, which perturb protein homeostasis and lead to endoplasmic reticulum (ER) stress. To relieve ER stress and restore proteostasis, tumor cells have adopted a specific stress response pathway, UPR, that includes three main branches initiated by IRE1α, PERK, and ATF6 [52]. The signaling pathways in the three branches cooperate to reduce protein translation and enhance the ER-folding capacity. Additionally, the ERAD pathway is activated to translocate and degrade the misfolded proteins accumulated in the ER. Notably, ERAD and UPR are engaged in a crosstalk, in which, UPR triggers ERAD, while impaired ERAD activates UPR [53]. FTS has been reported to induce ER stress in *MYC*-amplified cancer cells as indicated by upregulation of UPR markers [54]. Analysis of gene expression profiles in FTS-treated cancer cells points to the upregulation of stress response genes, such as ATF3 and ATF4 [55]. We demonstrated that inhibition of ERAD, a downstream cellular process triggered by UPR, enhanced FTS efficacy, suggesting that FTS likely induces UPR in the KRAS-driven PDAC tumor cells. Therefore, we examined UPR in these PDAC cells. Intriguingly, FTS treatment induced sustained UPR in all three PDAC cell lines, shown by increased levels of phospho-PERK (p-PERK), phospho-eukaryotic translation initiation factor 2α (p-eIF2α), and ATF4 (Fig. 3a, Supplementary Fig. S2).

EerI was reported to induce accumulation of misfolded proteins and UPR in cancer cells by blocking ERAD [56] and/or inhibiting Sec61 complex-mediated translocation of newly synthesized chaperones from cytosol to the ER [57]. To determine the effect of combined FTS-Eer1 treatment on UPR, we examined the mRNA levels of multiple UPR marker genes. Compared to treatment with FTS or EerI alone, combined treatment elicited UPR hyperactivation through IRE1α and PERK branches, as indicated by the dramatic increase in XBP1s, ATF4, CHOP, and GADD34 transcripts (Fig. 3b) [56, 58]. Notably, ATF4/CHOP signaling, known to induce apoptotic cell death [59–61], was significantly enhanced by the combined treatment.

Current data support proadaptive as well as proapoptotic roles for UPR. Homeostatic UPR signaling may promote tumor progression by supporting tumor cell survival and resistance to chemotherapy and radiation therapy [62, 63]. However, in cases where ER stress remains overwhelmingly unresolved, activation of a terminal UPR program will lead to cell death [64–66]. Apoptosis following ER stress can be mediated by multiple mechanisms [67–69]. In particular, hyperactivation of the PERK/eIF2α/ATF4 pathway induces apoptosis through the regulation of proapoptotic transcription factor CHOP and Bcl-2 family proteins [70–76]. Consistent with the upregulation of ATF4/CHOP upon combined treatment with FTS and EerI, significant apoptosis was observed in murine and human PDAC cell lines as detected by the Annexin V/7-AAD apoptosis assay (Fig. 2g-h). Thus, we show that combined treatment synergizes to induce apoptosis in PDAC tumor cells. Bcl-2 homology 3 (BH3)-only proapoptotic proteins, such as BIM and NOXA, are regulated by ATF4/CHOP signaling and mediate ER stress-induced apoptosis [56, 73, 77]. Indeed, BIM protein was significantly upregulated upon combination treatment in PDAC cells (Fig. 3c, Supplementary Fig. S2). Pretreatment of PDAC cells with a PERK inhibitor (PERKi), GSK2606414, partially blocked the induction of cell death by the combined treatment (Fig. 3d), suggesting that activation of PERK/eIF2α/ATF4/CHOP proapoptotic pathway contributes to the synergistic cell death.

### Genetic knockout of SYVN1 and SEL1L sensitizes PDAC cells to FTS treatment

**Fig. 4:**
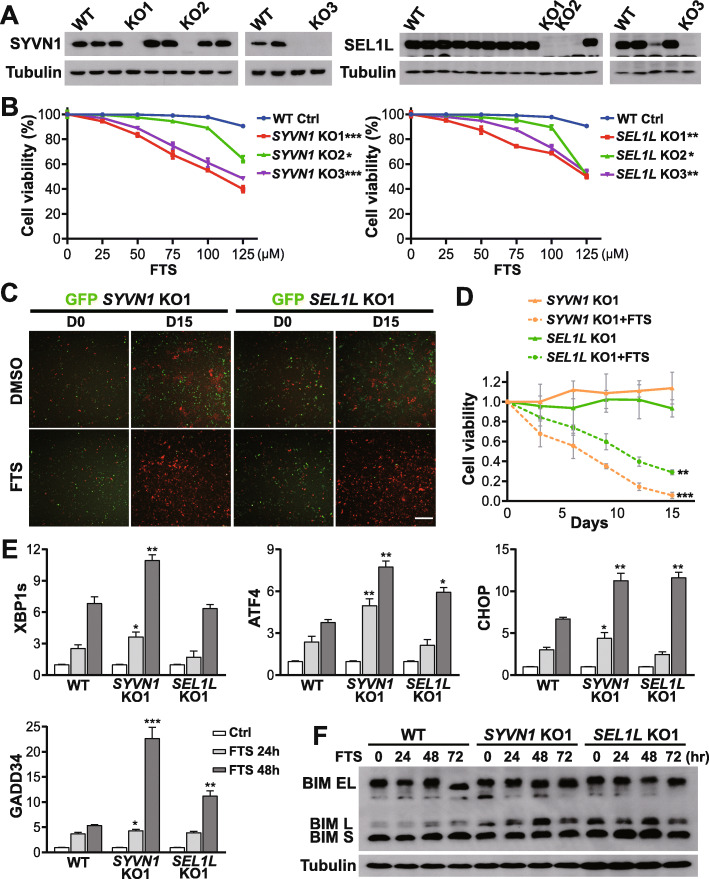
Knockout of *SYVN1* and *SEL1L* sensitizes PDAC cells to FTS treatment through proapoptotic UPR. **a** Validation of *SYVN1* and *SEL1L* CRISPR knockouts by western blot analysis of single clones derived from MIA PaCa-2 cells. **b** Relative cell viability of wild type control and *SYVN1* and *SEL1L* KO clones treated with 25-125 μM FTS for five days. Two-tailed student’s t test, *P < 0.05, **P < 0.01, ***P < 0.001; data are presented as mean ± SD; *n =* 3. **c** Representative images of multicolor competition assay (50X) in the presence of 125 μM FTS. Scale bar, 100 μm. **d** Quantitative analysis of MCA for *SYVN1* KO1 and *SEL1L* KO1 cell following FTS treatment. Unpaired two-sample t test, **P < 0.01, ***P < 0.001; data are presented as mean ± SD; *n =* 3. **e** Changes in transcript levels of UPR markers (XBP1s, ATF4, CHOP, and GADD34) in wild type control, *SYVN1* KO1 and *SEL1L* KO1 cells following treatment with 100 μM FTS for 48 h. All mRNA values were normalized to human GAPDH. Two-tailed student’s t test compares transcriptional difference between KO clones and WT control at the same timepoint, *P < 0.05, **P < 0.01, ***P < 0.001; data are presented as mean ± SD; *n =* 3. (F) BIM protein expression in wild type control, *SYVN1* KO1, and *SEL1L* KO1 cells following treatment with 100 μM FTS for 72 h

HRD1-SEL1L complex is an essential component of ERAD pathway. HRD1, encoded by *SYVN1* gene, is an E3 ubiquitin ligase involved in processing of misfolded proteins. SEL1L is an HRD1 adaptor protein, and indispensable for ERAD [78]. To demonstrate genetically that inhibition of ERAD indeed enhances the FTS therapeutic efficacy, we knocked out *SYVN1* and *SEL1L* genes in MIA PaCa-2 cells utilizing the CRISPR/Cas9 system. Complete loss of *SYVN1* and *SEL1L* expression was confirmed by Western blot analysis and genomic DNA sequencing (Fig. 4a, Supplementary Fig. S3–4). We tested three individual knockout (KO) clones for each gene (Fig. 4a) and demonstrated that these clones exhibited increased sensitivity to FTS treatment (Fig. 4b). To further examine the drug sensitivity in the knockout clones, we performed a multicolor competition assay (MCA), in which the mCherry-labeled wild type control clone was co-cultured with GFP-labeled *SYVN1* KO1 or *SEL1L* KO1 clones at a 1:1 ratio, and treated with either vehicle or FTS for 2 weeks [79]. Compared to the non-treated population, the FTS-treated cells showed predominance of mCherry-labeled wild type cells, indicating increased vulnerability of *SYVN1* and *SEL1L* deficient cells (Fig. 4c-d). Thus, PDAC cells with genetic defects in the ERAD machinery are more sensitive to FTS treatment.

**Fig. 5:**
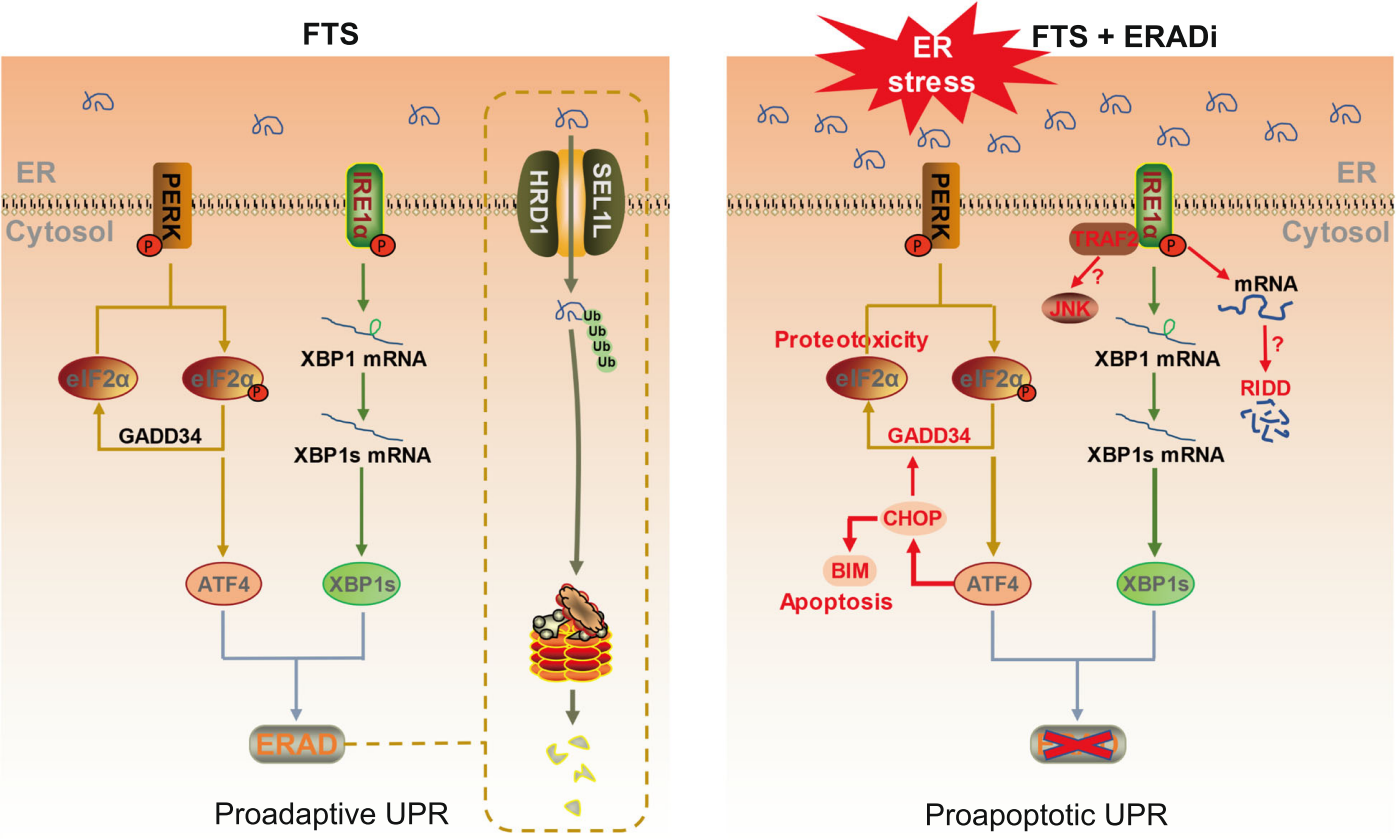
Schematic model of the synthetic lethal effects of FTS and ERAD inhibition. Following FTS treatment, UPR is drastically enhanced by ERAD inhibition, eliciting unresolved ER stress and apoptosis

In *SYVN1* and *SEL1L* KO cells, increased sensitivity to FTS coincided with increased level of ER stress. *SYVN1* KO1 and *SEL1L* KO1 cells treated with FTS showed dramatic upregulation of UPR marker genes, including XBP1s, ATF4, CHOP, and GADD34 (Fig. 4e), suggesting that cells with genetic defects in ERAD are more prone to proapoptotic UPR. Furthermore, expression of the pro-apoptotic protein BIM, in particular the BIM L isoform, was strongly induced in these clones following FTS treatment (Fig. 4f, Supplementary Fig. S4), pointing to the induction of apoptosis by the hyperactive ER stress. In summary, using both pharmacological and genetic approaches, we demonstrate that targeting ERAD improves the therapeutic efficacy of FTS through increased activation of ER stress and the subsequent induction of apoptosis (Fig. 5).

## Discussion

Using a genome-wide CRISPR synthetic lethal screen, we have identified ERAD as a key cellular target to enhance FTS efficacy. We show that FTS treatment induces ER stress, which is further amplified by inhibition of ERAD, resulting in synergistic induction of apoptosis in PDAC cells (Fig. 5). In pancreatic cancer, a highly desmoplastic stroma leads to hypoxia and nutrients deprivation [80]. Therefore, pancreatic cancer is more vulnerable to the induction of ER stress. Consequently, therapeutic options that promote ER stress and activate apoptosis may be exploited for PDAC treatment. We have identified one such therapeutic approach by combining FTS and ERAD inhibitors, the efficacy of which, may be examined in vivo for the treatment of PDAC. Similarly, FTS may be combined with other ER stress inducers, such as proteasome inhibitors [81], to generate severe ER stress and tumor cell apoptosis. Combined use of FTS and gemcitabine has been empirically examined for clinical pancreatic cancer treatment [22]. Our study on the mechanisms of drug synergy will provide new options for future PDAC treatments. Recent studies suggest that resolution of ER stress leads to tumor proliferation and formation of metastases from the disseminated PDAC cells [82]. Under constitutive ER stress, metastatic cells may remain dormant and fail to develop overt tumor masses. In this light, the combined use of FTS and ERAD inhibitors, may serve as a novel therapeutic option against metastatic PDAC.

Regulation of tumor cell survival following ER stress is complicated [62, 63, 83]. Our study argues that FTS-induced ER stress is relieved by activation of proadaptive UPR and ERAD, while ERAD inhibition through induction of ER stress, leads to hyperactivation of PERK/eIF2α/ATF4/CHOP/BIM proapoptotic pathway and massive cell death. Additionally, activation of IRE1α signaling pathway can recruit TRAF2 and JNK to induce cell death, or contribute to apoptosis through regulation of IRE1-dependent decay (RIDD) [84, 85]. On the other hand, proapoptotic mediators, such as ATF4 and CHOP, have been shown to activate multiple autophagy genes as a pro-survival response to counteract the ER stress [86]. Thus, cell fate control following ER stress may depend on the fine-tuning of UPR, and the crosstalk between UPR, ERAD, autophagy, and apoptosis [53, 87, 88].

Our CRISPR screen has identified additional targets that may enhance the FTS therapeutic efficacy. In a transgenic mouse model of pancreatic cancer, cell survival following KRAS ablation depends on mitochondrial function, especially oxidative phosphorylation [35]. In this regard, our study has identified several genes encoding mitochondria complex I subunits, mitochondria complex II subunits, as well as the mitochondrial iron-sulfur cluster assembly machinery [89–91]. Additionally, we identified Sptlc1, Sptlc2, and Kdsr genes, encoding the serine palmitoyltransferase (SPT) and 3-ketodihydrosphingosine reductase (KDHR), among the top candidates. SPT and KDHR serve as essential enzymes in the initial steps of de novo sphingolipid synthesis [92]. As sphingolipids are structural components of plasma membrane, inhibition of sphingolipid metabolism may mislocalize oncogenic KRAS and abolish its signaling [93]. Our findings suggest that multiple approaches to dislodge KRAS from plasma membrane may be combined to abrogate oncogenic KRAS signaling. Other candidate genetic targets in our screen are involved in apoptosis, mTOR signaling, and fatty acids and selenoprotein metabolism [94, 95]. Further studies will examine the role of these genetic targets in modulating FTS efficacy in the treatment of PDAC.

## Conclusion

Using both genetic and pharmacological approaches, we have demonstrated a critical role for ERAD in enhancing the FTS therapeutic response. The combined use of FTS and ERAD inhibitors may be an effective therapeutic option for the treatment of PDAC.

## Supplementary Information

**Additional file 1: Supplementary Fig. S1.** Combination indexes show synergistic effect between EerI and FTS (A) Berenbaum combination index (CI). CI value of lower than 1 indicates synergy. (B) Cell viability tests presented as mean ± SD. Data analysis and calculation of IC50 was performed using GraphPad Prism 8. (C) The CI values of FTS and EerI in 4292, PANC-1, and MIA PaCa-2 cells, indicating synergistic effect. **Supplementary Fig. S2.** Original images of the Western blot presented in Fig. 3a and c. **Supplementary Fig. S3.** Validation of *SYVN1* and *SEL1L* CRISPR knockouts by PCR and Sanger sequencing (A) Map of *SYVN1* and *SEL1L* and their respective sgRNAs. Half-arrows indicate the primer sets used to for PCR amplification. Primer sequences are listed in Supplementary Table 5. (B) PCR amplicons of wild type control and the knockouts using primer sets depicted in S3A. (C) Sanger sequencing of *SYVN1* exon3 PCR products. In *SYVN1* KO1, one allele shows 40 nt deletion between the targeting sites of sg2 and sg3, and mutations/frameshift around sg3 targeting site in the other allele. (D) Sanger sequencing of *SEL1L* 1F-3R PCR product from *SEL1L* KO1 validating a 106 nucleotide deletion between the targeting sites of sg1 and sg3. **Supplementary Fig. S4**. Original images of the Western blot presented in Fig. 4a and f

**Additional file 2: Supplementary Table 1.** Genes rank based on their beta scores in ascending order from the FTS screen. **Supplementary Table 2**. GO pathway analysis of top candidate genes from FTS screen. **Supplementary Table 3**. The sequences of primers used in qPCR assay. **Supplementary Table 4**. sgRNA oligonucleotide sequences for SYVN1/SEL1L knockout. **Supplementary Table 5.** PCR primers used to validate genomic knockout of SYVN1 and SEL1L

## Data Availability

CRISPR screen data is available at GEO accession number GSE162065.

## Abbreviations

PDAC: Pancreatic ductal adenocarcinoma
CRISPR: Clusters of regularly interspaced short palindromic repeats
FTS: Farnesyl thiosalicylic acid
EerI: Eeyarestatin I
UPR: Unfolded protein response
ERAD: Endoplasmic reticulum-associated protein degradation
IC50: Half-maximal inhibitory concentration
qPCR: Quantitative real-time PCR
GFP: Green fluorescent protein
MCA: Multicolor competition assay
GO: Gene Ontology
GSEA: Gene Set Enrichment Analysis
CI: Combination index
p-eIF2α: Phospho-eukaryotic translation initiation factor 2α
p-PERK: Phospho-PERK
PERKi: PERK inhibitor
KO: Knockout
SPT: Serine palmitoyltransferase
KDHR: 3-ketodihydrosphingosine reductase
