# Supplementary material for: Inhibition of ERAD synergizes with FTS to eradicate pancreatic cancer cells
Source: BMC Cancer. 2021 Mar 6;21:237. doi: 10.1186/s12885-021-07967-6 (PMC7937230; doi:10.1186/s12885-021-07967-6)

Inhibition of ERAD synergizes with FTS to eradicate pancreatic cancer cells

Rong Du[1,2,3], Delaney K. Sullivan[4], Nancy G. Azizian[1,2], Yuanhui Liu[1,2], Yulin Li[1,2*]

[1]Center for Immunotherapy Research, Houston Methodist Research Institute, Houston, TX 77030, USA; [2]Department of Medicine, Weill Cornell Medical College, New York, NY 10065, USA; [3]Department of Neurosurgery, Xiangya Hospital, Central South University, Changsha, Hunan 410008, China. [4]UCLA-Caltech Medical Scientist Training Program, David Geffen School of Medicine, University of California, Los Angeles, Los Angeles, CA 90095, USA

***Corresponding author**: Yulin Li, Houston Methodist Research Institute. 6670 Bertner Avenue. Houston, TX 77030, USA. Phone: 713-441-7350; Fax: 713-441-7438; Email: yli@houstonmethodist.org

List of supplementary figures

Supplementary Figure S1. Combination indexes show synergistic effect between EerI and FTS

(A) Berenbaum combination index (CI). CI value of lower than 1 indicates synergy.
(B) Cell viability tests presented as mean ± SD. Data analysis and calculation of IC50 was performed using GraphPad Prism 8.
(C) The CI values of FTS and EerI in 4292, PANC-1, and MIA PaCa-2 cells, indicating synergistic effect.

Supplementary Figure S2. Original images of the Western blot presented in Figure 3A and 3C

Supplementary Figure S3. Validation of *SYVN1* and *SEL1L* CRISPR knockouts by PCR and Sanger sequencing

(A) Map of *SYVN1* and *SEL1L* and their respective sgRNAs. Half-arrows indicate the primer sets used to for PCR amplification. Primer sequences are listed in **Supplementary Table 5**.
(B) PCR amplicons of wild type control and the knockouts using primer sets depicted in **S3A**.
(C) Sanger sequencing of *SYVN1* exon3 PCR products. In *SYVN1* KO1, one allele shows 40nt deletion between the targeting sites of sg2 and sg3, and mutations/frameshift around sg3 targeting site in the other allele.
(D) Sanger sequencing of *SEL1L* 1F-3R PCR product from *SEL1L* KO1 validating a 106 nucleotide deletion between the targeting sites of sg1 and sg3.

Supplementary Figure S4. Original images of the Western blot presented in Figure 4A and 4F

Supplementary Figure S1

A

$$CI = \frac{D_{FTS}}{IC50_{FTS}} + \frac{D_{EerI}}{IC50_{EerI}} \begin{cases} >1 \text{ for antagonism} \\ = 1 \text{ for additivity} \\ <1 \text{ for synergy} \end{cases}$$

$IC50_{FTS}$ – IC50 of FTS alone

$IC50_{EerI}$ – IC50 of EerI alone

D_{FTS} – IC50 of FTS in the presence of $IC50_{EerI}$

D_{EerI} – IC50 of EerI in the presence of $IC50_{FTS}$

B

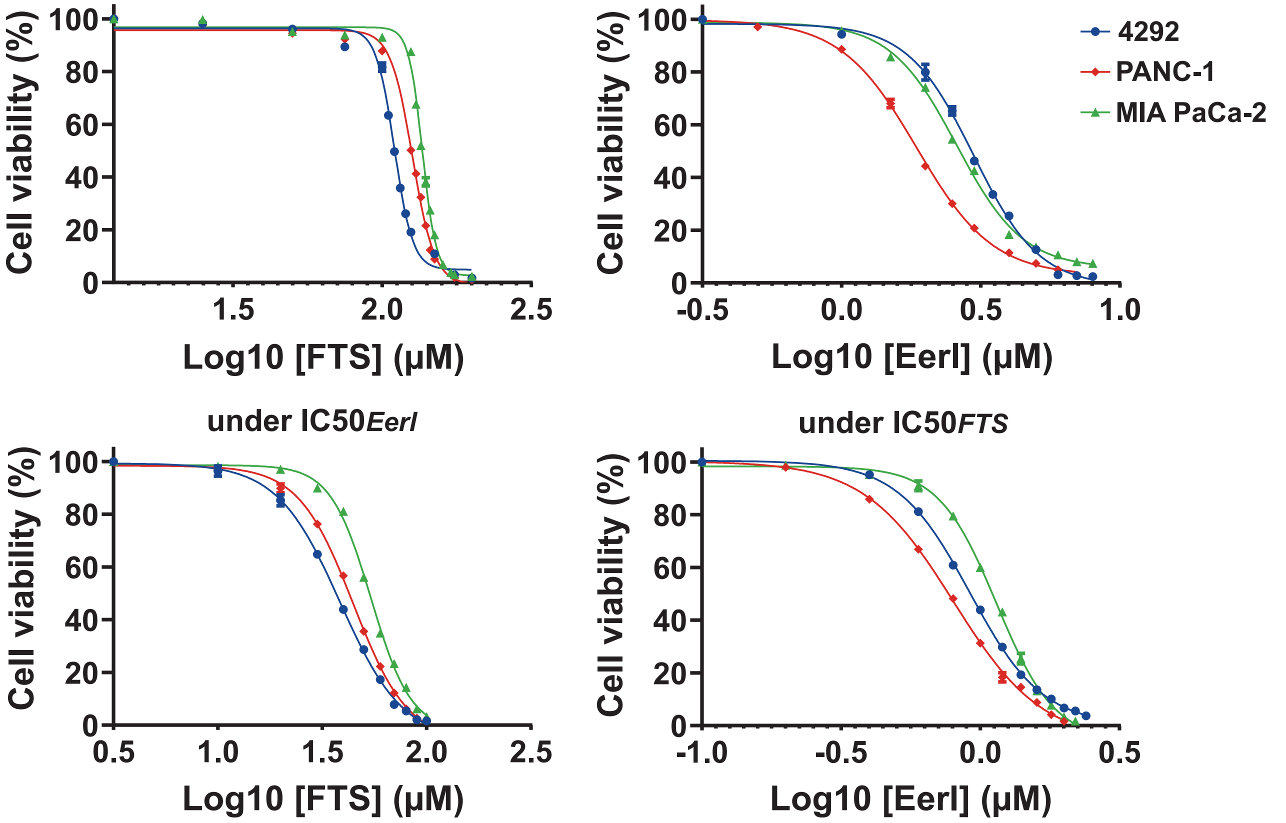

C

	$IC50_{FTS}$ (μM)	$IC50_{EerI}$ (μM)	D_{FTS} (μM)	D_{EerI} (μM)	CI
4292	110.0	2.982	38.56	0.920	0.659
PANC-1	126.5	1.863	44.55	0.802	0.783
MIA PaCa-2	136.5	2.595	54.15	1.146	0.838

Supplementary Figure S2

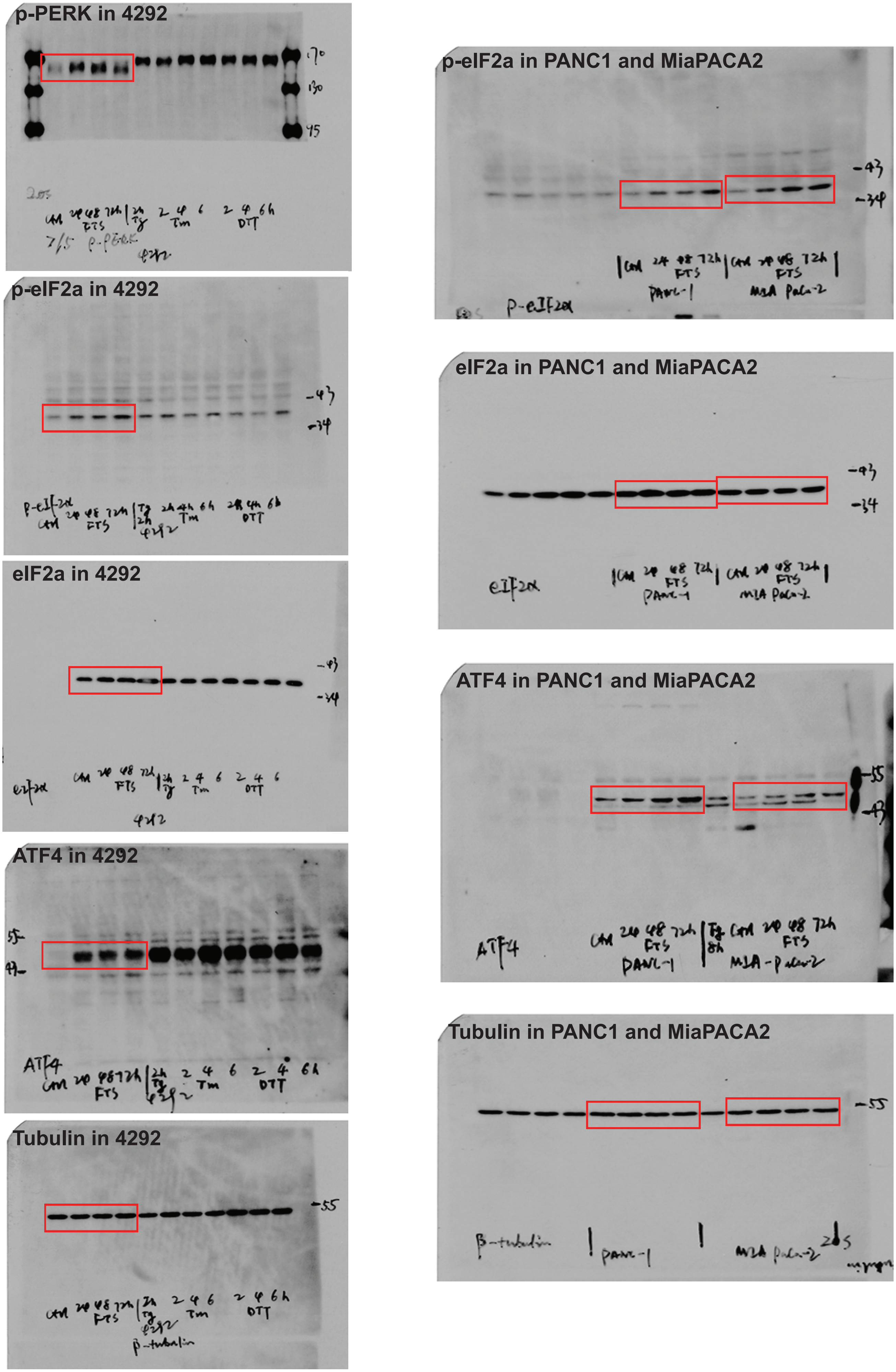

Supplementary Figure S2 (Continued)

Blots in Figure 3C

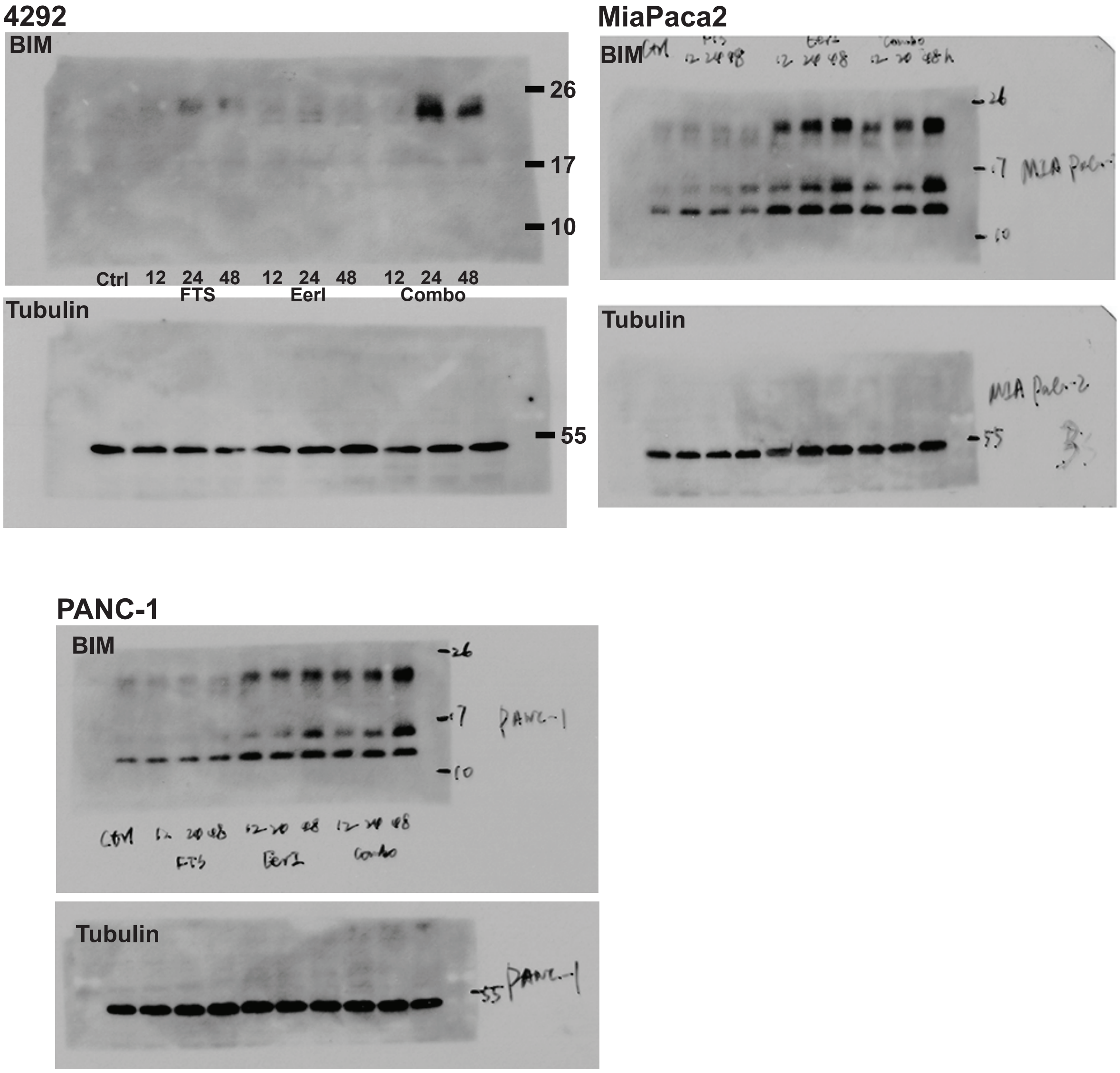

Supplementary Figure S3

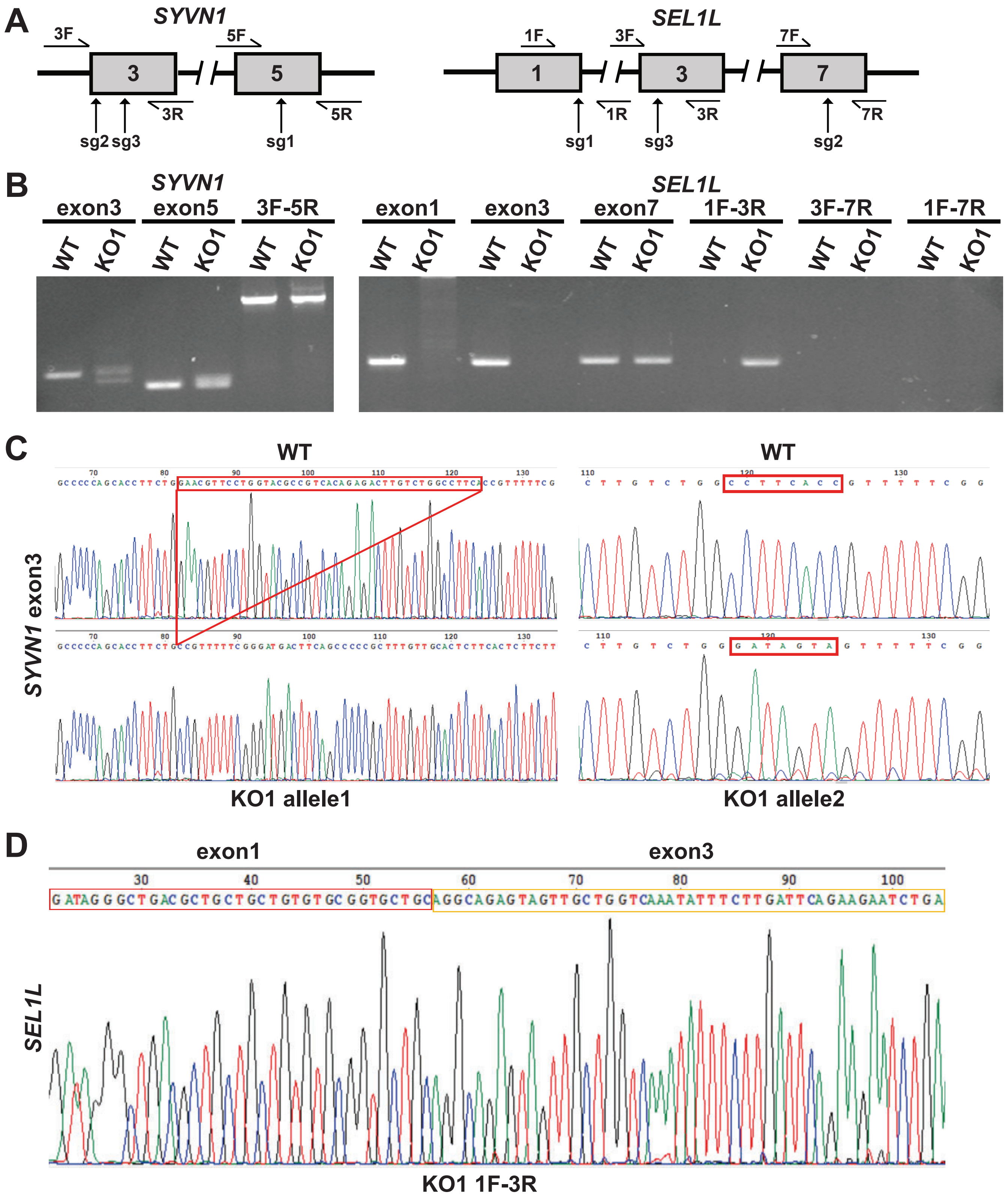

Supplementary Figure S4

Blots in Figure 4A

SYVN1 (KO1 and KO2)

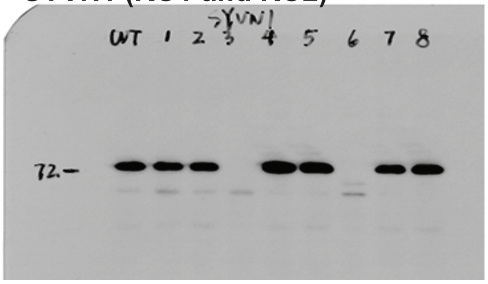

SEL1L

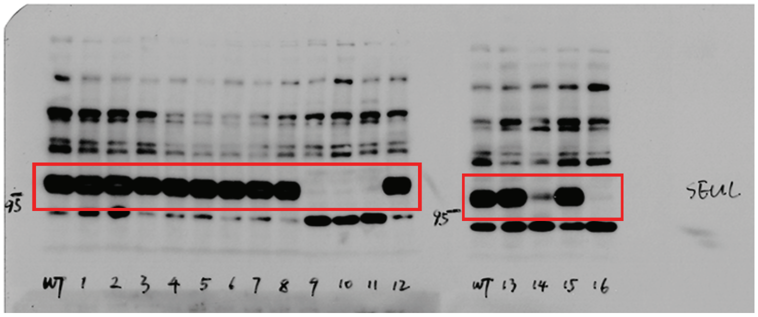

Tubulin

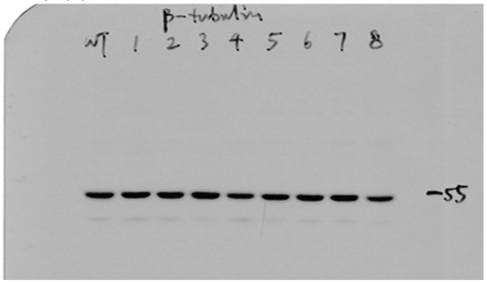

Tubulin

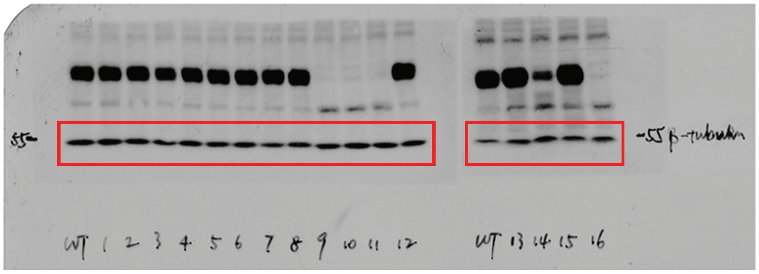

SYVN1 (KO3)

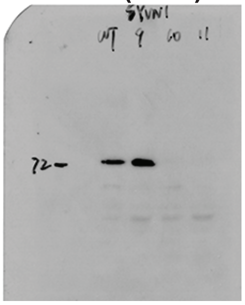

Tubulin

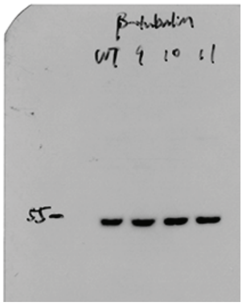

Blots in Figure 4F

BIM, Tubulin

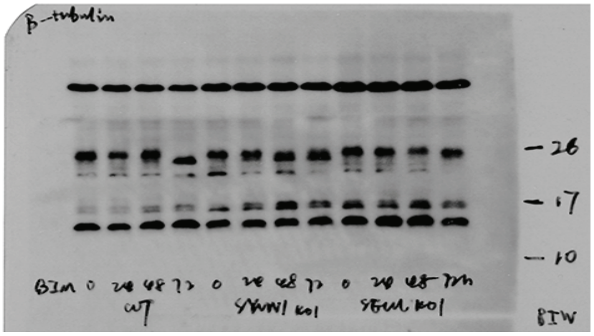

Supplement: Supplementary file 1 — Additional file 1: Supplementary Fig. S1. Combination indexes show synergistic effect between EerI and FTS (A) Berenbaum combination index (CI). CI value of lower than 1 indicates synergy. (B) Cell viability tests presented as mean ± SD. Data analysis and calculation of IC50 was performed using GraphPad Prism 8. (C) The CI values of FTS and EerI in 4292, PANC-1, and MIA PaCa-2 cells, indicating synergistic effect. Supplementary Fig. S2. Original images of the Western blot presented in Fig. 3a and c. Supplementary Fig. S3. Validation of SYVN1 and SEL1L CRISPR knockouts by PCR and Sanger sequencing (A) Map of SYVN1 and SEL1L and their respective sgRNAs. Half-arrows indicate the primer sets used to for PCR amplification. Primer sequences are listed in Supplementary Table 5. (B) PCR amplicons of wild type control and the knockouts using primer sets depicted in S3A. (C) Sanger sequencing of SYVN1 exon3 PCR products. In SYVN1 KO1, one allele shows 40 nt deletion between the targeting sites of sg2 and sg3, and mutations/frameshift around sg3 targeting site in the other allele. (D) Sanger sequencing of SEL1L 1F-3R PCR product from SEL1L KO1 validating a 106 nucleotide deletion between the targeting sites of sg1 and sg3. Supplementary Fig. S4. Original images of the Western blot presented in Fig. 4a and f [file 12885_2021_7967_MOESM1_ESM.pdf]
